# Usefulness of metabolic score for insulin resistance to predict restenosis after coronary stent implantation

**DOI:** 10.1080/07853890.2026.2679841

**Published:** 2026-06-09

**Authors:** Jie Tang, Panpan Xu, Jun Guo, Panpan Hao

**Affiliations:** a State Key Laboratory for Innovation and Transformation of Luobing Theory, Key Laboratory of Cardiovascular Remodeling and Function Research of MOE, NHC, CAMS and Shandong Province, Department of Cardiology, Qilu Hospital of Shandong University, Jinan, Shandong, China; b Department of Cardiology, Wuzhong People’s Hospital, Ningxia Medical University, Wuzhong, Ningxia, China

**Keywords:** Metabolic score for insulin resistance, in-stent restenosis, percutaneous coronary intervention

## Abstract

**Objective:**

The aim of this study was to evaluate the clinical forecasting potential of the Metabolic Score for Insulin Resistance (METS-IR) as a reliable predictor for in-stent restenosis (ISR) among patients receiving percutaneous coronary intervention (PCI).

**Methods:**

A total of 818 patients undergoing repeat coronary angiography for recurrent chest pain after percutaneous coronary intervention from 2022 to 2025 were retrospectively collected. Clinical, blood and angiography-related data were analyzed. The predictive efficacy of METS-IR was assessed through both univariate and multivariate logistic regression analyses, along with receiver operating characteristic curves, for the evaluation of ISR (defined as 50% luminal narrowing) and severe ISR (defined as 70% luminal narrowing).

**Results:**

There was a significant correlation between METS-IR and ISR. Patients with ISR >50% had higher METS-IR (42.30 ± 7.54 vs. 40.17 ± 6.31, *p* < 0.001), as did those with ISR >70% (43.05 ± 7.84 vs. 40.19 ± 6.29, *p* < 0.001). ISR prevalence increased with progressively across METS-IR tertiles (*p* < 0.001). Multivariate analysis confirmed METS-IR as an independent predictor of ISR (OR 1.28 for ISR >50%, 1.51 for ISR >70%, *p* < 0.05). ROC analysis identified an optimal METS-IR cutoff of 42.27 for ISR >50% and ISR >70% (sensitivity 50%, specificity 63%). Furthermore, incorporating the tertiles of METS-IR into the fully adjusted model significantly enhanced the ability to discriminate ISR.

**Conclusion:**

Our findings established that elevated METS-IR levels were strongly correlated with an increased risk of in-stent restenosis, with a markedly pronounced impact on severe ISR (>70%). METS-IR can serve as a straightforward and practical predictor for risk stratification after coronary stent implantation.

## Graphical abstract

**Figure:**
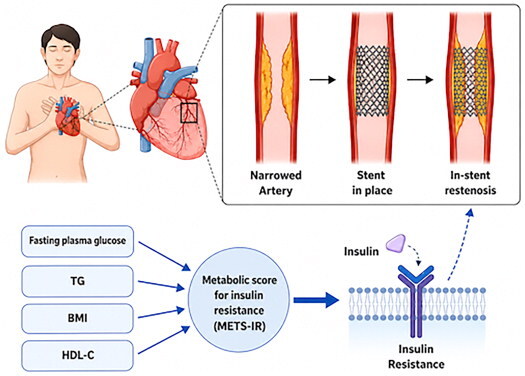

## Introduction

Coronary atherosclerotic heart disease, particularly acute myocardial infarction, remains a major contributor to global mortality [1,2], leading to adverse cardiovascular-related outcomes [3]. Although the development and refinement of percutaneous coronary intervention (PCI) and drug therapies significantly enhanced survival rates and improved clinical outcomes [4], the unresolved problem of in-stent restenosis (ISR) remains a critical limitation to treatment efficacy [5–7]. Therefore, identifying and researching the risk factors and formation mechanisms of in-stent restenosis is an important clinical step in preventing and treating in-stent restenosis. Neointimal hyperplasia and neointimal atherosclerosis are one of the main pathological processes of restenosis after coronary stent implantation [8]. Research has shown that reendothelialization in the stent and persistent inflammation continue to impede optimal stent function [9]. Emerging evidence suggests that complement activation is involved in restenosis pathogenesis [10]. At the same time, there is increasing evidence that insulin resistance (IR) also contributes to ISR pathogenesis through multiple pathways [11].

Insulin resistance (IR) represents fundamental pathophysiologic mechanism underlying systemic inflammatory processes and metabolic syndrome (MetS) [12,13], Metabolic dysfunction acts as a central pathophysiological process, simultaneously driving β-cell failure in type 2 diabetes mellitus (DM) and promoting atherosclerotic progression in CHD *via* chronic inflammation and endothelial dysfunction [14,15], Emerging clinical evidence further demonstrates that IR actively participates in the advancement of hypertensive disorders and early-onset coronary artery disease [16,17], Moreover, accumulating research indicates that IR may exert substantial effects on both the initiation and progression of in-stent restenosis (ISR) [18,19]. Conventional quantitative techniques to assess IR are limited by cost, time and labor, whereas novel biomarkers overcome these traditional methodological limitations [20], emerging as clinically viable alternatives that have predictive power for cardiovascular disease [21,22]. However, clinical evidence illustrating the relationship between METS-IR and ISR remains scarce. Hence, we designed the retrospective study of patients with recurrent chest pain after percutaneous coronary intervention to evaluate the potential association between ISR and METS-IR, providing an evaluation of METS-IR as a possible risk indicator for ISR in post-PCI patients.

## Methods

### Data source and study population

The clinical information for this retrospective cohort study was obtained from the electronic medical record. The study adhered to the ethical standards of the Declaration of Helsinki. Ethical approval was granted by the Ethics Committee of Qilu Hospital (approval number KYLL-2025-11-064), Shandong University, before data collection, and written informed consent was secured from all participants. Patients who developed recurrent chest pain after PCI and required repeat angiography were prospectively identified from January 2022 to February 2025. Patients were included from the study inclusion criteria: (1) age ≥18 years; (2) a prior history of PCI within 6-72 months and accompanied by repeat coronary angiography; and (3) patients capable of providing documented informed consent voluntarily. Patients were excluded from the study based on the following conditions: (1) prior coronary artery bypass surgery; (2) lesions managed solely with balloon angioplasty in the absence of stent placement; (3) clinical manifestations of heart failure, structural cardiomyopathies, congenital cardiac anomalies, or significant valvular pathology; (4) severe hepatic or renal dysfunction (defined as eGFR <30 mL/min/1.73 m^2^); (5) active systemic inflammatory processes, malignant neoplasms, hematologic abnormalities, or autoimmune conditions; and (6) insufficient clinical documentation for analysis. After applying all selection criteria, 818 cases met the eligibility criteria and were included in the following analyzes.

### Definitions and the process of data collection

Patient data included demographic characteristics (age, gender, BMI, systolic blood pressure and diastolic blood pressure, smoking and drinking status), related medical history (including hypertension, diabetes, dyslipidemia, previous stroke), medication use (angiotensin-converting enzyme inhibitors (ACEI) or angiotensin receptor blockers (ARB), aspirin, clopidogrel, ticagrelor, beta-blockers, dual antiplatelet therapy (DAPT), statin and dapagliflozin), partial laboratory data including TC, TG, High-density lipoprotein cholesterol(HDL-C) and Low-density lipoprotein cholesterol(LDL-C), FPG, Albumin(ALB), Uric acid(UA), Serum creatinine(SCr), eGFR and LVEF) and key coronary angiography parameters. All data materials are derived from a retrospective collection of electronic health records. The calculation of METS-IR was determined as follows: Ln [(2 × FPG (mg/dL)) + fasting TG (mg/dL)] × BMI (kg/m^2^) ÷ Ln [HDL-C (mg/dL)].

### Evaluation of the ISR

All study patients underwent standardized follow-up coronary angiography *via* the Judkins method following their PCI procedure. Traditionally, In-stent restenosis was confirmed angiographically according to established criteria, defined as >50% luminal narrowing either within the stent or within the 5-mm adjacent segments [23]. The broader concept of ISR is further divided into angiographic and clinical ISR. Angiographic ISR is traditionally defined as *a* > 50% diameter stenosis, while clinical ISR refers to *a* > 50% stenosis accompanied by recurrent symptoms or ischemic, or *a* > 70% stenosis even if asymptomatic [24–26]. The investigation employed angiographic criteria for patient stratification in light of missing functional assessment parameters and quantitative coronary measurements. We respectively conducted the evaluation for ISR (defined as 50% luminal narrowing) and severe ISR (defined as 70% luminal narrowing), which may help accurately assess the clinical significance of restenosis. To investigate whether insulin resistance, as quantified by the METS-IR index, serves as an independent predictor for the occurrence of ISR development following PCI, the METS-IR index was categorized into tertiles: Tertile 1 (METS-IR <37.89), Tertile 2 (37.89 ≤METS-IR <43.56) and Tertile 3 (METS-IR ≥43.56).

### Statistical analysis

Statistical analyses were performed using SPSS 27.0 and R 4.2.1. Continuous variables were summarized as mean ± SD or median (IQR) and were compared using independent t-tests or Mann-Whitney U tests, respectively. Categorical variables were expressed as frequencies (%) and analyzed using the chi-square test or Fisher’s exact test.

Potential predictors of ISR were first evaluated through univariate logistic modeling. Variables with a *p*-value of 0.05 in the univariate analysis as well as clinically relevant disease and treatment characteristics, were included in the multivariable models. To evaluate the independent association between METS-IR (analyzed as both continuous and categorical variables) and ISR, progressively adjusted multivariable logistic regression models were developed. Variance inflation factors (VIFs) were computed to assess multicollinearity, with VIF < 10 indicating no significant collinearity among covariates. Results are expressed as odds ratios (ORs) with 95% confidence intervals (CIs).

Stratified analyses were performed by sex, smoking status, alcohol consumption, hypertension, diabetes, dyslipidemia and previous stroke, with interaction effects assessed by multiplicative interaction terms. To evaluate the diagnostic efficacy of METS-IR, receiver operating characteristic (ROC) curve analysis was employed to calculate the area under the curve (AUC) and identify the ideal threshold. The strength of association was reported as Odds ratio (ORs) with 95% confidence interval (CI). Finally, the incremental predictive value of the model was compared by evaluating model 3 with and without the inclusion of METS-IR. A p-value threshold of 0.05 was established for statistical significance.

## Results

### Baseline clinical characteristics

**Figure 1.**
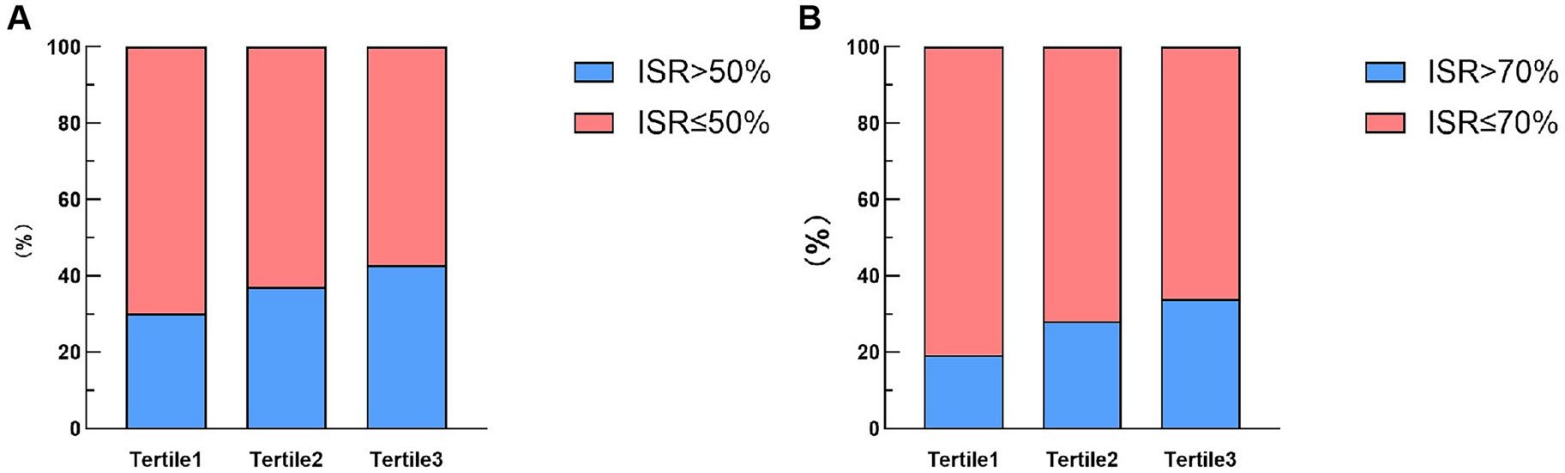
The effects of METS-IR on the prevalence of patients with an ISR >50% (A) and an ISR >70% (B) in three groups based on the tertiles of METS-IR.

The study cohort comprised 818 participants, with an average age 62.41 ± 9.56. Table 1 and S1 show the study population stratified by tertiles of METS-IR (Tertile 1: *n* = 269, METS-IR <37.89; Tertile 2: *n* = 278, 37.89 ≤METS-IR <43.56; Tertile 3: *n* = 271, METS-IR ≥43.56), with clinical characteristics compared between these groups. As the METS-IR level increased, the proportion of ISR (including ISR >50% and ISR >70%) among different groups gradually increased (Figure 1). The key baseline characteristics of patients stratified by tertiles of the METS-IR index are shown in Table 1, and additional baseline characteristics are presented in Supplementary Table S1. Compared to the lower tertile, patients in the upper tertile demonstrated significantly higher rates of male, current smokers, and alcohol consumers. Furthermore, this group showed elevated incidences for hypertension, diabetes, and dyslipidemia. Regarding laboratory examination, the levels of triglyceride (TG), LDL-C, FPG, uric acid (UA) and serum creatinine (Scr) showed a positive correlation with increasing METS-IR tertiles, while HDL-C level was inversely correlated.

**Table 1.** Key baseline characteristics of patients stratified by tertiles of the METS-IR index.

Variables	Tertile1 (*n* = 269)	Tertile2 (*n* = 278)	Tertile3 (*n* = 271)	*p*-value
METS-IR	33.75 ± 3.09	40.68 ± 1.69	48.42 ± 4.72	
Age (years)	63.84 ± 8.94	63.36 ± 9.30	60.03 ± 9.99	<0.001
Male, n (%)	147 (54.6)	207 (74.5)	211 (77.9)	<0.001
BMI (kg/m^2^)	22.92 ± 1.96	25.92 ± 1.66	28.77 ± 2.76	<0.001
Risk factors, n (%)				
Smoking, n (%)	81 (30.1)	107 (38.5)	116 (42.8)	0.008
Drinking, n (%)	65 (24.2)	98 (35.3)	115 (42.4)	<0.001
Hypertension, n (%)	150 (55.8)	194 (69.8)	215 (79.3)	<0.001
Diabetes, n (%)	110 (40.9)	125 (45.0)	145 (53.5)	0.011
Dyslipidemia, n (%)	152 (56.5)	212 (76.3)	251 (92.6)	<0.001
Previous stroke, n (%)	23 (8.6)	34 (12.2)	30 (11.1)	0.363
Laboratory examination				
TC (mg/dl)	128.82 ± 34.91	130.04 ± 37.70	126.88 ± 35.10	0.584
TG (mg/dl)	89.46 (66.43-113.81)	108.06 (80.38-146.14)	134.63(104.51-194.85)	<0.001
HDL-C (mg/dl)	46.82 ± 9.92	40.06 ± 7.76	34.51 ± 6.80	<0.001
LDL-C (mg/dl)	67.05 ± 27.52	73.25 ± 29.49	73.22 ± 28.38	0.014
UA (μmol/l)	285.06 ± 68.20	308.08 ± 78.17	324.18 ± 84.47	<0.001
FPG (mg/dl)	89.64 (82.08-100.80)	97.02 (85.77-114.30)	102.06 (90.90-127.08)	<0.001
eGFR (mL/min/1.73 m^2^)	91.46 ± 17.72	91.22 ± 19.42	93.32 ± 22.00	0.400
LVEF (%)	62 (57-66)	62 (57-66.25)	62 (57-65)	0.592
Angiographic characteristics				
Multi-vessel lesion, n (%)	124 (45.42)	129 (47.25)	125 (45.96)	0.907
Stent number	2.36 ± 0.54	2.34 ± 0.54	2.34 ± 0.54	0.284
Stent length (mm)	27.00 (20.00-33.00)	26.00 (20.00-33.00)	29.00 (22.00-33.00)	0.077
Stent diameter (mm)	2.75 (2.50-3.00)	3.00 (2.50-3.00)	3.00 (2.75-3.00)	0.096

Notes: Data are presented as mean ± SD, median (IQR), or n (%), as appropriate. A two-sided *p* < 0.05 was considered statistically significant. Additional baseline characteristics, including medication use and detailed angiographic/procedural characteristics, are presented in Supplementary Table S1.

BMI: body mass index; eGFR: estimated glomerular filtration rate; FPG: fasting plasma glucose; HDL-C: high-density lipoprotein cholesterol; LDL-C: low-density lipoprotein cholesterol; LVEF: left ventricular ejection fraction; METS-IR: metabolic score for insulin resistance; SD: standard deviation; TC: total cholesterol; TG: triglyceride; UA: uric acid.

**Figure 2.**
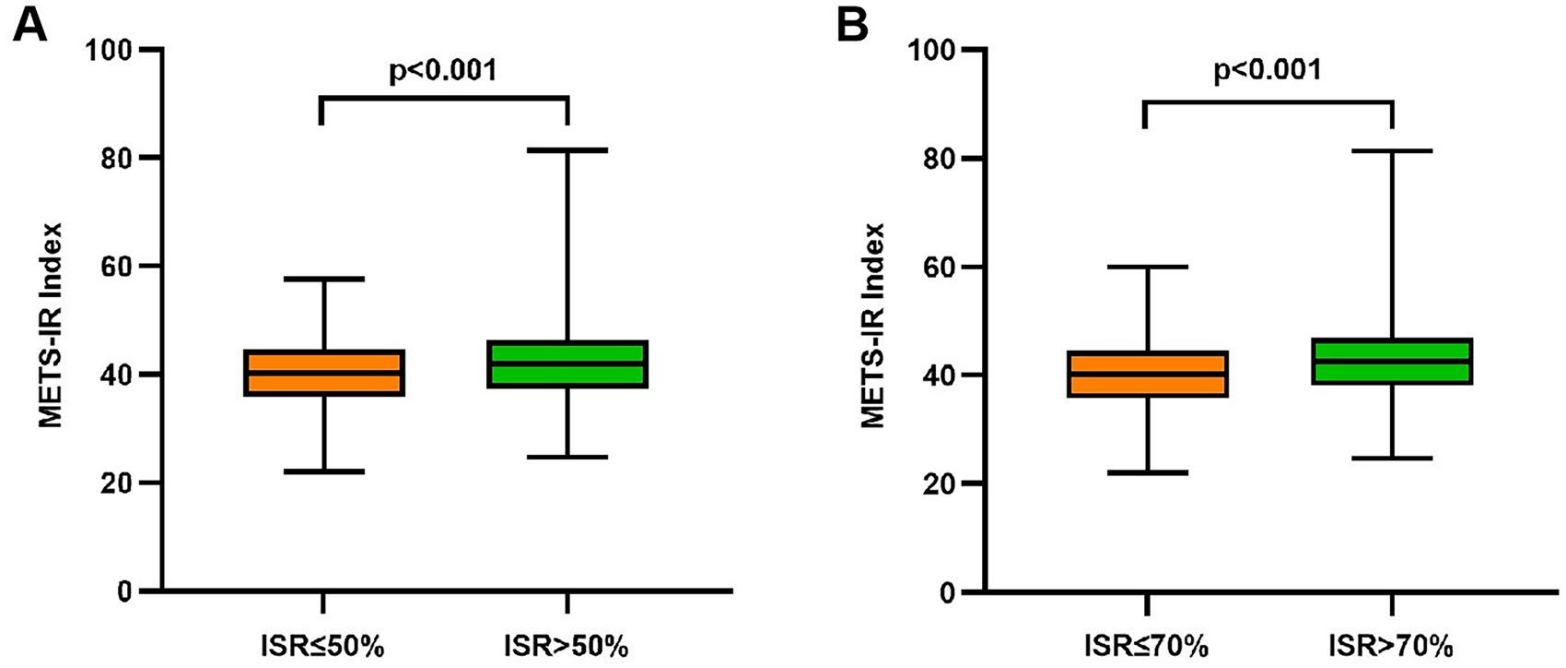
Comparison of METS-IR values between patients with ISR >50% and those with ISR≤ 50% (A) and comparison of METS-IR values between the patients with ISR >70% and those with ISR ≤ 70% (B).

Based on the angiographic threshold of 50% luminal narrowing, patients were stratified into two groups: ISR >50% (*n* = 300) and ISR ≤50% (*n* = 518). As presented in Table S2, comprehensive baseline demographic, clinical, biochemical parameters and angiographic characteristics were compared across groups. Notably, the ISR >50% group demonstrated a significantly greater burden of several cardiovascular risk factors than the ISR ≤50% group, including higher proportions of current smoking, alcohol consumption, hypertension and diabetes alongside increased utilization of hypoglycemic medications (all *p* < 0.05). Regarding laboratory parameters, total cholesterol (TC), triglycerides (TG), LDL-C, FPG and serum creatinine (SCr) also differed significantly between the two groups with *p* < 0.05. Angiographic characteristics showed significantly increased in stent-related characteristics, including stent number, length and diameter, within the ISR >50% group relative to the ISR ≤50% group. More importantly, compared with patients exhibiting ≤50% ISR, the METS-IR values were substantially elevated in the group with ISR >50% (42.30 ± 7.54 vs 40.17 ± 6.31, *p* < 0.001), indicating a strong association between insulin resistance and the severity of restenosis (Figure 2A).

For more detailed analysis, we categorized patients into the severe ISR group (>70%, *n* = 222) and the non-severe ISR group (≤70%, *n* = 596). Consistent with the analysis of the threshold of 50% luminal narrowing, Table S3 demonstrates a similar trend. Patients with severe ISR (>70%) exhibited a significantly higher prevalence of diabetes and a corresponding increase in hypoglycemic medication use. Laboratory examination revealed significantly higher levels of TC, TG, LDL-C, FPG and SCr, coupled with reduced HDL-C levels, in the severe ISR group (>70%) with all *p* < 0.05. Angiographic characteristics also revealed significant increases in stent-related parameters with stent number, length and diameter in the severe group (>70%), compared to non-severe group. There was no significant difference between the two groups in terms of multivessel disease (121(54.50) vs. 129 (47.25), *p* = 0.921) and target vessel location (LM-LAD, LCX and RCA, both *p* > 0.05). Moreover, the METS-IR values were substantially elevated in patients with ISR >70% group than in ISR ≤70% (43.05 ± 7.84 vs. 40.19 ± 6.29, *p* < 0.001) (Figure 2B).

### Correlational analysis of METS-IR with conventional cardiovascular indicators

Table 2 shows the correlations between METS-IR and several traditional cardiovascular indicators. Positive correlation with BMI, gender, uric acid, serum creatinine (*r* = 0.854, 0.211, 0.239, 0.117, respectively; all *p* < 0.001) and LDL-C (*r* = 0.083, *p* = 0.018). Conversely, a significant negative correlation was observed with age (r = −0.202, *p* < 0.001). Regression analysis revealed no significant relationship between METS-IR and total cholesterol or homocysteine levels.

**Table 2.** Association between METS-IR index and other cardiometabolic risk factors.

Variables	Correlation coefficient	*p*-value
Age, years	−0.202	**<0.001**
BMI	0.854	**<0.001**
Gender	0.211	**<0.001**
UA, mg/dl	0.239	**<0.001**
LDL, mg/dl	0.083	**0.018**
TC, mg/dl	−0.034	0.335
SCr, μmol/l	0.117	**<0.001**
df	Reference	1

Notes: The *p*-values in bold are all <0.05.

BMI: body mass index; LDL-C: low-density lipoprotein-cholesterol; UA: uric acid; LDL: low-density lipoprotein-cholesterol; TC: total cholesterol; SCr: Serum creatinine.

### Univariate and multivariate associations between METS-IR and ISR risk

Univariate logistic regression analysis was conducted to identify potential predictors of ISR (Table S4). When analyzed as a continuous variable, each standard deviation (SD) increment in METS-IR was significantly linked to an increased risk of both ISR >50% (OR = 1.37, 95% CI: 1.18–1.58, *p* < 0.001) and ISR >70% (OR = 1.46, 95% CI: 1.25–1.71, *p* < 0.001). Furthermore, univariate analysis also identified several significant predictors, including BMI, diabetes, FPG, LDL-C, SCr, beta-blocker use, clopidogrel therapy, stent length, and stent diameter for both ISR >50% and ISR >70%.

Multivariate logistic regression analyses showed that an increase in METS-IR levels was positively correlated with the likelihood of ISR occurrence, even after adjusting for confounding factors (Tables 3 and S5). Modeled as a continuous variable, each standard deviation (SD) increased in METS-IR significantly associated with ISR >50% across all models (Model 1: OR = 1.36; Model 2: OR = 1.32; Model 3: OR = 1.28; all *p* < 0.01). Similarly, the risk of ISR >70% increased with each SD increment in METS-IR: The adjusted OR (95% CI) was 1.57 (1.33–1.86) in Model 1, 1.55 (1.25–1.85) in Model 2 and 1.42 (1.18–1.71) in Model 3 (all *p* < 0.001).

**Table 3.** Significant predictors of ISR >70%in multivariate logistic regression analyses.

METS-IR index	OR (95% CI)		
	Model 1	Model 2	Model 3
Per unit increase	1.07 (1.04–1.09)**	1.07 (1.04–1.09)**	1.05 (1.02–1.08)**
Per SD increase	1.57 (1.33–1.86)**	1.55 (1.25–1.85)**	1.42 (1.18–1.71)**
Tertile 1	1 Reference	1 Reference	1 Reference
Tertile 2	1.61 (1.07–2.42)*	1.56 (1.04–2.36)*	1.46 (0.94–2.26)
Tertile 3	2.21 (1.48–3.34)**	2.10 (1.39–3.20)**	1.75 (1.12–2.75)*
*P* for trend	**<0.001**	**<0.001**	**0.015**

Model 1: adjusted for age, gender.

Model 2: adjusted for variables with a *p*-value < 0.05 in the univariate analysis, including, smoking, drinking, hypertension, previous stroke as well as age and gender.

Model 3: adjusted for age, gender, smoking, drinking, hypertension, diabetes, previous stroke, coronary angiography interval, aspirin, total cholesterol, low-density lipoprotein cholesterol, uric acid, LM-LAD lesion, RCA lesion, stent length and stent diameter.

* *p* < 0.05.

^**^ *p* < 0.01.

The *p*-values in bold are all <0.05.

Furthermore, categorical analysis demonstrated a significantly higher risk of ISR > 50% the adjusted OR (95% CI) was 1.68 (1.17–2.42) in model 1 (*p* = 0.006), 1.53 (1.05–2.23) in model 2 (*p* = 0.026). For ISR >70%, with the lowest tertile as the reference, patients in the highest tertile exhibited approximately a twofold increase in risk across Model 1 (OR = 2.21, 95% CI: 1.48–3.34), Model 2 (OR = 2.10, 95% CI: 1.39–3.20), and Model 3 (OR = 1.75, 95% CI: 1.12–2.75; all *p* < 0.05). Multivariate logistic regression analyses consistently identified METS-IR as an independent predictor of both ISR >50% and ISR >70%, irrespective of whether it was modeled as a continuous or categorical variable (Table 3).

### Receiver operating characteristic curves

**Figure 3.**
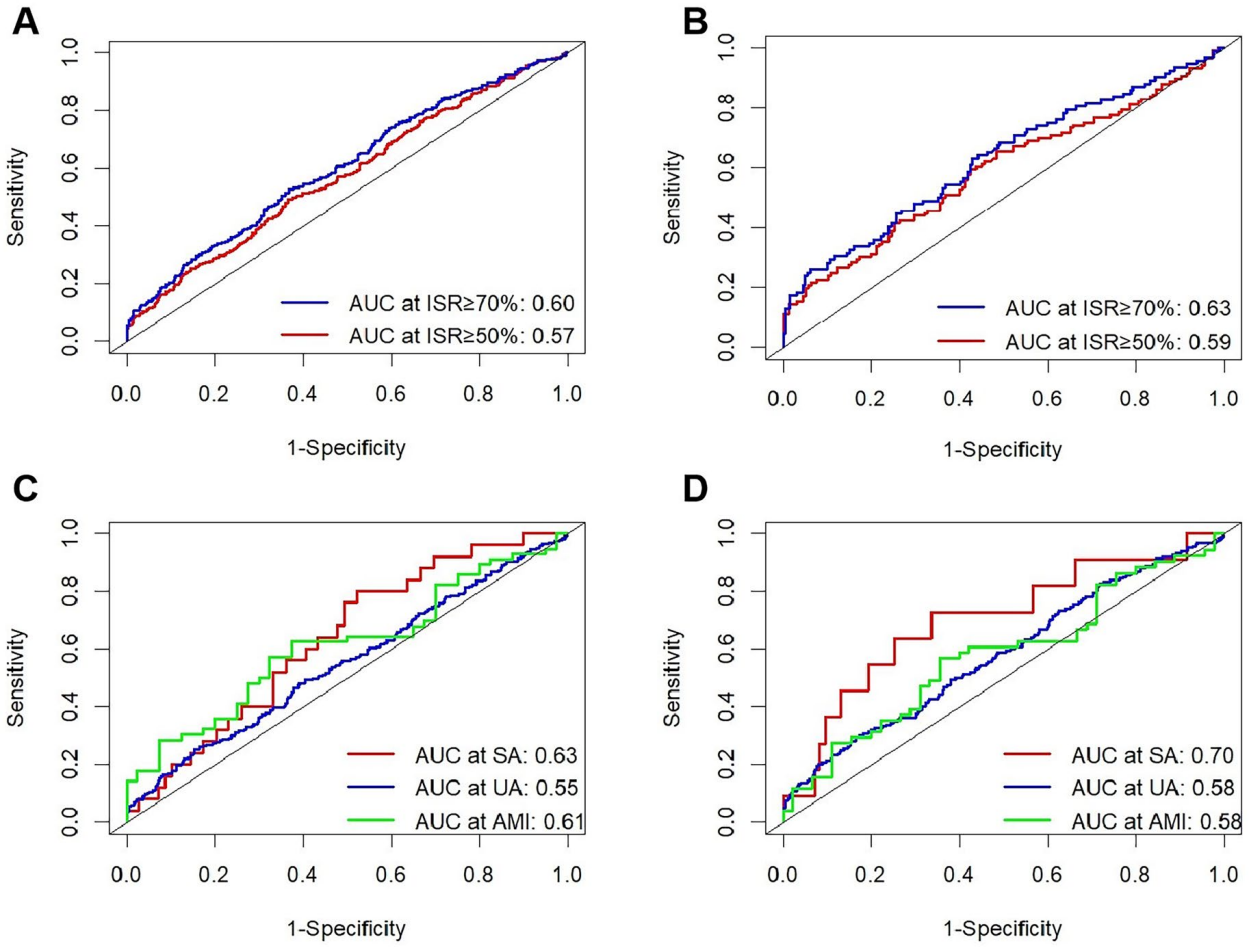
Receiver operating characteristic (ROC) curve analysis of METS-IR for prediction of ISR >50% and ISR >70% (A) in all patients, and ROC curve analysis of METS-IR for prediction of ISR >50% and ISR >70% (B) in patients with high METS-IR, and ROC curve analysis of METS-IR for prediction of ISR >50% (C) in patients with stable angina, unstable angina and acute myocardial infarction, and ROC curve analysis of METS-IR for prediction of ISR >70% (D) in patients with stable angina, unstable angina and acute myocardial infarction.

We used ROC curve analysis evaluated the predictive capacity of METS-IR for ISR. For ISR >50%, METS-IR demonstrated moderate discriminative ability (AUC = 0.57, 95% CI: 0.53-0.61, *p* < 0.001), with an optimal threshold of 42.27 providing balanced sensitivity (49.0%) and specificity (63.3%). Similar predictive performance was observed for ISR >70% (AUC = 0.60, 95% CI: 0.56-0.64, *p* < 0.001), where the identical cut-off value (42.27) showing slightly improved sensitivity (52.5%) while maintaining comparable specificity (63.0%) (Table S6; Figure 3A).

Then, we assessed the predictive performance of METS-IR among the patients with elevated baseline METS-IR levels. ROC analysis revealed the optimal cut-off value of 46.41 for predicting ISR >50% in this high-value group (AUC = 0.59, 95% CI: 0.52–0.66; *p* = 0.011; sensitivity: 65.5%, specificity: 51.6%). Notably, discriminatory power improved when predicting the severe ISR (>70%) (AUC = 0.63, 95% CI: 0.55–0.70; *p* = 0.001). The cutoff increased slightly to 46.80, yielding 63.7% sensitivity and 55.6% specificity (Table S6; Figure 3B).

In addition, we conducted ROC analysis on METS-IR to examine the discriminative ability of METS-IR in predicting in-stent restenosis in patients with different types of coronary heart disease after PCI (Table S6; Figure 3C and 3D). METS-IR demonstrated the highest predictive value in patients with stable angina, particularly for >70% ISR (AUC = 0.70, 95% CI: 0.53–0.87, *p* = 0.033), with an optimal cutoff of 39.75 (sensitivity: 72.7%, specificity: 66.3%). Its predictive value for >50% ISR in this subgroup was moderate (AUC = 0.63, *p* < 0.05). In patients with unstable angina, the predictive utility was modest for both >50% and >70% ISR (AUCs of 0.55 and 0.58, respectively; both *p* < 0.05). Notably, METS-IR exhibited no significant predictive value for either ISR threshold in patients with acute myocardial infarction.

### Incremental effects of METS-IR on the predictive value of ISR

As shown in Tables 4 and S7, and Figure 3A), METS-IR demonstrated moderate predictive value for both >50% and >70% ISR (AUC = 0.57, 95% CI: 0.53–0.61; and AUC = 0.60, 95% CI: 0.56–0.64, both *p* < 0.001, respectively), with both thresholds demonstrating optimal discrimination at a METS-IR value of 42.27. What’s more, incorporating METS-IR into established risk assessment models substantially optimized the prediction of ISR. Specifically, for ISR >70%, reclassification analyses further supported these findings, showing significant net reclassification improvement (NRI = 0.29, *p* < 0.001) for moderate stenosis and superior integrated discrimination (IDI = 0.02, *p* < 0.001) for severe cases. Model fit statistics, including the C-statistic, AIC and BIC, confirmed these improvements across both stenosis severity thresholds.

**Table 4.** Assessment of the goodness-of -fit of models.

Comparison	Model 3 without METS-IR	Model 3 with METS-IR	*p*-value
Continuous NRI (95%CI)	Reference	0.29 (0.14–0.45)	**<0.001**
IDI (95%CI)	Reference	0.02 (0.01–0.03)	**<0.001**
C-statistic (95%CI)	0.71 (0.67–0.75)	0.72 (0.68–0.76)	–
AIC	901.4	889.3	–
BIC	981.5	974.1	–
df	Reference	1	

Notes: The *p*-values in bold are all <0.05.

METS-IR: metabolic score for insulin resistance; AIC: Akaike information criterion; BIC: Bayesian information criterion; df: degree of freedom.

### Subgroup analysis

**Figure 4.**
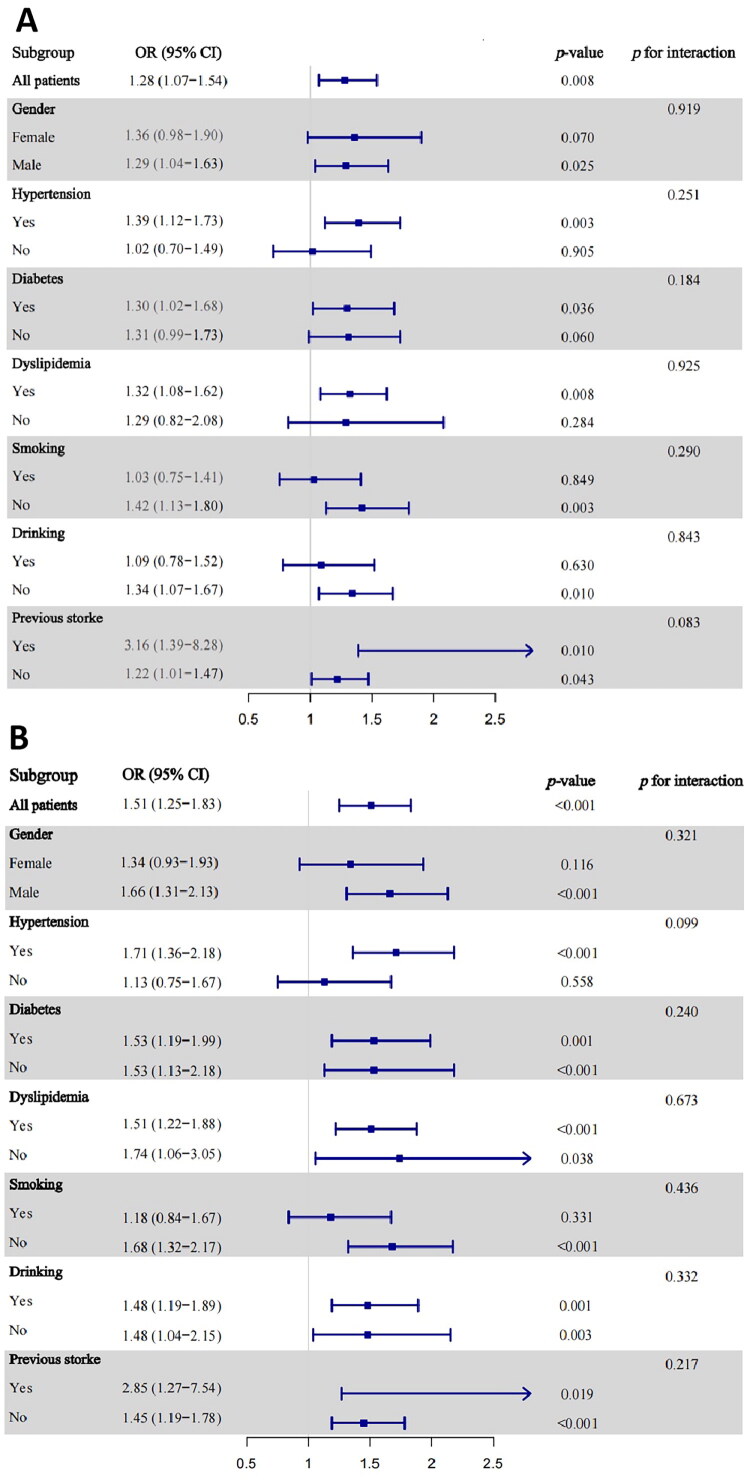
** **A subgroup analysis was performed to assess the association between METS-IR and ISR >50% (A) and ISR >70% (B), stratified by gender, alcohol consumption, smoking status, diabetes, dyslipidemia, hypertension and history of stroke.

Subsequent subgroup analysis evaluations were executed to confirm the capacity of METS-IR to anticipate in-stent restenosis, including ISR group (>50%) and severe ISR group (>70%). Categorizing participants based on gender, lifestyle and comorbidities demonstrated a significantly magnified predictive efficacy of METS-IR specifically in men, those with hypertension, dyslipidemia, and diabetes. In the fully adjusted model, no significant interaction was observed between METS-IR and any of the subgroups in relation to ISR >50% and ISR >70%. These results demonstrate that METS-IR maintains a stable risk prediction ability in different characteristic populations (Figure 4A and 4B).

## Discussion

Previous studies had shown that insulin resistance can lead to various diseases [27,28], and promote the development of cardiovascular disease mainly through various complex molecular mechanisms such as metabolic abnormalities, inflammatory response, endothelial dysfunction, oxidative stress and vascular remodeling [29]. Insulin resistance promotes atherosclerosis by causing disturbances in glucose and lipid metabolism. The mechanism includes compensatory hyperinsulinemia, which promotes proliferation of vascular smooth muscle cells and leads to thickening of blood vessel walls; glucotoxicity: Hyperglycemia promotes the formation of glycosylation end products (AGEs), exacerbates vascular inflammation and hardening. Evidence indicates that insulin resistance independently contributes to the pathogenesis of diverse cardiovascular disorders [30,31] and has also been associated with the development of coronary atherosclerosis [32,33].

Traditional insulin resistance indices, such as HOMA-IR and QUICKI, have been widely used for the assessment of insulin resistance [34]. Compared with several traditional insulin resistance indices, METS-IR may have several practical and clinical advantages. By integrating multiple metabolic parameters, including fasting plasma glucose, body mass index, high-density lipoprotein cholesterol, and triglycerides, METS-IR may provide a more comprehensive assessment of insulin resistance and overall metabolic status than individual metabolic parameters. More importantly, all variables required for calculating METS-IR are routinely available in daily clinical practice and can be obtained without additional insulin measurements or complex testing, making this index inexpensive, convenient, and potentially suitable for routine clinical use and large-scale risk screening. Emerging evidence has shown that METS-IR, as a simple and readily obtainable indicator, is associated with several cardiovascular and metabolic disorders, including hypertension, diabetes, heart failure, atrial fibrillation, and recurrence after radiofrequency ablation [35–39]. However, its predictive value for in-stent restenosis after PCI, particularly severe restenosis, remains insufficiently investigated.

In this study, we investigated the association between the METS-IR and the risk of ISR after percutaneous coronary intervention. Several important findings emerged. Our analysis first indicates that climbing METS-IR METS-IR values tightly correlate with an amplified risk of ISR, a finding consistent across both continuous and tertile-based evaluations. Secondly, we observed a markedly heightened occurrence of ISR in the high-METS-IR-score cohort when contrasted with the low-score group. Third, METS-IR levels were correlated with established cardiovascular risk factors and remained independently associated with ISR risk after adjustment for potential confounders. Notably, the predictive value of METSIR appeared to be more pronounced when ISR was defined as >70% luminal stenosis than >50% luminal stenosis. These findings imply that severe instances of restenosis (ISR >70%) are more heavily driven by metabolic dysregulation. Together, these results identify METS-IR as an independent predictor of ISR and suggest its potential value in improving current risk stratification for patients undergoing PCI.

Consistent with these findings, statistical modeling *via* multivariate logistic regression confirmed the independent prognostic value of METS-IR for ISR. The comprehensive subgroup analysis demonstrated that this association remained consistent across various patient characteristics, including sex, tobacco use, alcohol intake, and comorbid conditions such as hypertension, diabetes, dyslipidemia, and previous stroke events.

Interestingly, our study demonstrated a distinct predictive pattern of METS-IR across different clinical presentations, with a stronger predictive value observed in patients with stable angina pectoris than in other groups. This difference may be attributed to the distinct pathophysiological mechanisms underlying ISR in these populations. In patients with stable angina, ISR is mainly driven by chronic neointimal hyperplasia, a gradual process that is closely associated with systemic insulin resistance and metabolic dysfunction – factors directly reflected by METS-IR. The results also suggests that METS-IR could function as a valuable clinical tool for identifying patients at the risk of stent restenosis, especially with a higher degree of restenosis within the stent.

Additionally, patients in the restenosis group in our study exhibited larger stent-related parameters, including longer stent length, greater stent diameter, and a higher number of implanted stents compared with the non-restenosis group, which is consistent with previous reports [40]. This finding may be attributed to the fact that more complex or extensive coronary lesions often require longer or multiple stents, leading to a larger stented segment and increased vascular injury, thereby facilitating neointimal hyperplasia and increasing the risk of restenosis [41,42].

In contemporary clinical practice, revascularization decisions require comprehensive assessment of clinical presentation, coronary anatomy, ischemic burden, comorbidities, procedural risk, and expected long-term benefit [43]. Established risk tools, such as the GRACE score and SYNTAX/SYNTAX II score, have been widely used to support risk stratification and individualized treatment selection, including conservative therapy, PCI, or CABG in appropriate clinical settings [44,45]. These examples indicate that risk scores can provide clinically meaningful information beyond technical feasibility alone.

In this context, METS-IR should be regarded as a complementary metabolic risk marker rather than a standalone decision-making tool. Elevated METS-IR may help identify patients at higher risk of ISR after PCI and provide additional information for individualized risk stratification and postoperative management. Clinically, such patients may benefit from more intensive control of modifiable metabolic risk factors, optimized secondary prevention strategies, and closer follow-up after PCI. Even when PCI is ultimately performed, METS-IR may still help identify high-risk individuals who require stricter metabolic management and closer surveillance to reduce the risk of restenosis.

Several limitations must be acknowledged in this study. First, its retrospective, single-center study with a relatively limited sample size may introduce selection and measurement bias and limit the generalizability of our findings. Second, due to the limitations of the retrospective study, we were unable to control the time intervals of the follow-up. Third, ISR was evaluated solely *via* angiography, lacking the morphological precision of intravascular imaging (IVUS/OCT). Although METS-IR effectively reflects insulin resistance, we did not evaluate other established indices, limiting the ability to directly benchmark its predictive efficacy against alternative metabolic indicators. Future multicenter prospective studies integrating alternative metabolic indices and advanced intracoronary imaging (IVUS/OCT) are essential to validate these findings and elucidate the underlying mechanisms of METS-IR-driven ISR.

## Conclusions

Research indicates that higher levels of the METS-IR index may contribute to a greater likelihood of developing in-stent restenosis after PCI, particularly in patients with severe restenosis (ISR> 70%). By demonstrating the important involvement of insulin resistance in the development of ISR, proposing METS-IR as a straightforward and practical predictor to evaluate restenosis risk among patients undergoing PCI.

## Supplementary Material

Supplemental Material

## Data Availability

The datasets used and/or analyzed during the current study are available from the corresponding author on reasonable request.
