# Supplementary material for: Usefulness of metabolic score for insulin resistance to predict restenosis after coronary stent implantation
Source: Ann Med. 2026 Jun 9;58(1):2679841. doi: 10.1080/07853890.2026.2679841 (PMC13250870; doi:10.1080/07853890.2026.2679841)
Supplement: Supplemental Material [file IANN_A_2679841_SM8511.docx]

**Table S1. Additional baseline characteristics of patients stratified by tertiles of the METS-IR index**

| **Variables** | **Tertile1 (n=269)** | **Tertile2 (n=278)** | **Tertile3 (n=271)** | ***p*-value** |
| --- | --- | --- | --- | --- |
| **Blood pressure** |  |  |  |  |
| SBP (mmHg) | 129.51±17.52 | 131.99±19.01 | 132.18±16.58 | 0.103 |
| DBP (mmHg) | 74.06±10.43 | 75.66±11.04 | 76.60±10.36 | 0.020 |
| **Additional laboratory examination** |  |  |  |  |
| SCr (μmol/l) | 69.08±14.55 | 73.32±15.82 | 74.07±18.56 | <0.001 |
| ALB (g/l) | 43.60 (41.60-46.00) | 43.80 (41.88-46.20) | 44.20 (42.00-45.90) | 0.550 |
| **Medications, n (%)** |  |  |  |  |
| Beta-blockers | 145 (53.9) | 160 (57.6) | 156 (57.6) | 0.612 |
| ACEI/ARB | 71 (26.4) | 105 (37.8) | 102 (37.6) | 0.006 |
| Aspirin | 257 (95.5) | 266 (95.7) | 259 (95.6) | 0.996 |
| Clopidogrel | 197 (73.2) | 216 (77.7) | 181 (66.8) | 0.016 |
| Ticagrelor | 54 (20.1) | 48 (17.3) | 69 (25.5) | 0.057 |
| DAPT | 240 (89.2) | 254 (91.4) | 238 (87.8) | 0.394 |
| Statin | 258 (95.9) | 261 (93.9) | 249 (91.9) | 0.148 |
| Dapagliflozin | 48 (17.8) | 60 (21.6) | 66 (24.4) | 0.179 |
| **Detailed angiographic characteristics** |  |  |  |  |
| LM-LAD, n (%) | 210 (76.92) | 207 (75.82) | 223 (81.99) | 0.178 |
| LCX, n (%) | 46 (16.85) | 54 (19.78) | 39 (14.34) | 0.239 |
| RCA, n (%) | 151 (55.31) | 154 (56.41) | 145 (53.31) | 0.762 |
| Multi-vessel lesion, n (%) | 124 (45.42) | 129 (47.25) | 125 (45.96) | 0.907 |
| Stent number | 2.36 ± 0.54 | 2.34±0.54 | 2.34 ±0.54 | 0.284 |
| Stent length (mm) | 27.00 (20.00-33.00) | 26.00 (20.00-33.00) | 29.00 (22.00-33.00) | 0.077 |
| Stent diameter (mm) | 2.75 (2.50-3.00) | 3.00 (2.50-3.00) | 3.00 (2.75-3.00) | 0.096 |
| Bare metal stent, n (%) | 13 (4.8) | 16 (5.8) | 15 (5.5) | 0.883 |
| Drug-eluting stent, n (%) | 257 (95.5) | 262 (94.2) | 257 (94.8) | 0.790 |
| Coronary angiography interval  (months) | 24.00 (8.00-50.00) | 24.00(7.00-48.00) | 24.00(9.00-55.00) | 0.492 |
| Coronary artery disease, n (%) |  |  |  |  |
| SA | 46 (17.1) | 24 (8.6) | 24 (8.9) | 0.003 |
| UA | 204 (75.8) | 218 (78.4) | 206 (76.0) | 0.726 |
| AMI | 19 (7.1) | 36 (12.9) | 41 (15.1) | 0.011 |

Notes: Data are presented as mean ± SD, median (IQR), or n (%), as appropriate. A two-sided *p* < 0.05 was considered statistically significant.

Abbreviations: ACEI, angiotensin-converting enzyme inhibitor; ALB, albumin; AMI, acute myocardial infarction; ARB, angiotensin receptor blocker; DAPT, dual antiplatelet therapy; DBP, diastolic blood pressure; LCX, left circumflex artery; LM-LAD, left main-left anterior descending artery; METS-IR, metabolic score for insulin resistance; RCA, right coronary artery; SA, stable angina; SBP, systolic blood pressure; SCr, serum creatinine; SD, standard deviation; UA, unstable angina.

**Table S2. Baseline characteristics of the study population grouped with ISR >50% versus ISR≤ 50%**

| **Variables** | **Total (n=818)** | **ISR ≤50% (n=518)** | **ISR >50% (n=300)** | ***P*-value** |
| --- | --- | --- | --- | --- |
|  |  |  |  |  |
| Age (years) | 62.41±9.56 | 62.37±9.69 | 62.48±9.51 | 0.852 |
| Male, n (%) | 565 (69.1) | 344 (66.4) | 221 (73.7) | 0.030 |
| BMI (kg/m^2^) | 25.88±3.22 | 25.62±2.99 | 26.28±3.62 | 0.002 |
| SBP (mmHg) | 131.24±17.76 | 130.70±18.17 | 132.28±17.35 | 0.254 |
| DBP (mmHg) | 75.45±10.66 | 75.15±10.43 | 75.99±11.19 | 0.298 |
| **Risk factors, n (%)** |  |  |  |  |
| Smoking, n (%) | 304 (37.2) | 179 (34.6) | 125 (41.7) | 0.043 |
| Drinking, n (%) | 278 (34.0) | 160 (30.9) | 118 (39.3) | 0.014 |
| Hypertension, n (%) | 559 (68.3) | 339 (65.4) | 220 (73.3) | 0.019 |
| Diabetes, n (%) | 380 (46.5) | 224 (43.2) | 156 (52.0) | 0.016 |
| Dyslipidemia, n (%) | 615 (75.2) | 382 (73.7) | 233 (77.7) | 0.211 |
| Previous stroke, n (%) | 87 (10.6) | 56 (10.8) | 31 (10.3) | 0.831 |
| **Laboratory examination** |  |  |  |  |
| TC (mg/dl) | 128.59±35.93 | 125.95±34.83 | 133.23±37.54 | 0.006 |
| TG (mg/dl) | 108.50 (80.60-147.91) | 103.02 (79.71-139.05) | 119.13 (84.14-174.0) | <0.001 |
| HDL-C (mg/dl) | 40.44±9.65 | 40.86±9.62 | 40.06±9.75 | 0.107 |
| LDL-C (mg/dl) | 71.20±28.60 | 68.70±27.03 | 75.31±30.89 | <0.001 |
| UA (μmol/l) | 305.84±78.81 | 301.73±76.31 | 311.09±82.28 | 0.108 |
| FPG (mg/dl) | 95.76 (85.68-112.68) | 93.96 (84.92-110.03) | 98.46 (87.66-118.22) | 0.004 |
| SCr (μmol/l) | 72.17±16.55 | 70.40±15.09 | 75.24±18.37 | <0.001 |
| eGFR (ml/min/1.73 m^2^) | 92.00±19.79 | 93.46±18.98 | 89.47±20.91 | 0.005 |
| ALB (g/l) | 43.80 (41.80-46.00) | 44.00 (42.00-45.90) | 43.70 (41.53-46.10) | 0.565 |
| LVEF (%) | 62 (57-66) | 62 (57-66) | 62 (57-66) | 0.301 |
| **Medications, n (%)** |  |  |  |  |
| Beta-blockers, n (%) | 461 (56.4) | 310 (59.8) | 151 (50.3) | 0.008 |
| ACEI/ARB, n (%) | 278 (34.0) | 175 (33.8) | 103 (34.3) | 0.873 |
| Aspirin, n (%) | 782 (95.6) | 493 (95.2) | 289 (96.3) | 0.436 |
| Clopidogrel, n (%) | 594 (72.6) | 413 (79.7) | 181 (60.3) | <0.001 |
| Ticagrelor, n (%) | 171 (20.9) | 78 (15.1) | 93 (31.0) | <0.001 |
| DAPT, n (%) | 732 (89.5) | 469 (90.5) | 263 (87.7) | 0.197 |
| Statin, n (%) | 768 (93.9) | 492 (95.0) | 276 (92.0) | 0.086 |
| Dapagliflozin, n (%) | 174 (21.3) | 72 (13.9) | 102 (34.0) | <0.001 |
| **Angiographic characteristics** |  |  |  |  |
| LM-LAD, n (%) | 640 (78.24) | 396 (76.45) | 244 (81.33) | 0.123 |
| LCX, n (%) | 139 (16.99) | 81 (15.64) | 58 19.33) | 0.208 |
| RCA, n (%) | 450 (55.01) | 284 (54.83) | 166 (55.33) | 0.946 |
| Multi-vessel lesion, n (%) | 378 (46.21) | 228 (44.02) | 150 (50.00) | 0.114 |
| Stent number | 2.33 ± 0.54 | 2.29±0.54 | 2.39 ±0.54 | 0.010 |
| Stent length (mm) | 27.00 (22.00-33.00) | 24.00 (20.00-32.00) | 30.00 (25.00-36.00) | <0.001 |
| Stent diameter (mm) | 3.00 (2.50-3.00) | 2.75 (2.50-3.00) | 3.00 (2.75-3.00) | <0.001 |
| Bare metal stent, n (%) | 44 (5.4) | 22 (4.2) | 22 (7.3) | 0.059 |
| Drug-eluting stent, n (%) | 776 (94.9) | 497 (95.9) | 279 (93.0) | 0.066 |
| Coronary angiography interval  (months) | 24.00 (8.00-52.00) | 17.50 (6.00-48.00) | 36.00 (12.00-60.00) | <0.001 |
| **Coronary artery disease, n (%)** |  |  |  |  |
| SA | 94 (11.5) | 70 (13.5) | 24 (8.3) | 0.026 |
| UA | 628 (76.8) | 409 (79.0) | 219 (73.0) | 0.052 |
| AMI | 96 (11.7) | 40 (7.7) | 56 (18.7) | <0.001 |
| Index |  |  |  |  |
| METS-IR | 40.97±6.86 | 40.17±6.31 | 42.30±7.54 | <0.001 |

Notes: Data are presented as mean ± SD, median (IQR), or n (%), as appropriate. A two-sided p < 0.05 was considered statistically significant.

Abbreviations: BMI, body mass index; ACEI, angiotensin-converting enzyme inhibitors; ARB, angiotensin receptors; DAPT, dual antiplatelet therapy; DBP, diastolic blood pressure; TC, total cholesterol; TG, triglyceride; FPG, fasting blood glucose; HDL-C, high-density lipoprotein cholesterol; ISR, in-stent restenosis; LDL-C, low-density lipoprotein-cholesterol; LVEF, left ventricular ejection fraction; SBP, systolic blood pressure; UA, uric acid; SCr, creatinine; ACEI, angiotensin-converting enzyme inhibitors; ARB, angiotensin receptor blockers; SA, stable angina; UA, unstable angina; AMI, acute myocardial Infarction; METS-IR, metabolic score for insulin resistance.

**Table S3 Baseline characteristics of the study population grouped with ISR >70% versus ISR ≤ 70%**

| **Variables** | **Total (n=818)** | **ISR≤ 70% (n=596)** | **ISR >70% (n=222)** | **P-value** |
| --- | --- | --- | --- | --- |
|  |  |  |  |  |
| Age (years) | 62.41±9.56 | 62.21±9.62 | 62.95±9.38 | 0.328 |
| Male, n (%) | 565 (69.1) | 405 (68.0) | 160 (72.1) | 0.257 |
| BMI (kg/m^2^) | 25.88±3.22 | 25.64±2.98 | 26.52±3.73 | 0.002 |
| SBP (mmHg) | 131.24±17.76 | 131.01±18.30 | 131.85±16.24 | 0.524 |
| DBP (mmHg) | 75.45±10.66 | 75.30±10.56 | 75.85±10.93 | 0.507 |
| **Risk factors, n (%)** |  |  |  |  |
| Smoking, n (%) | 304 (37.2) | 217 (36.4) | 87 (39.2) | 0.464 |
| Drinking, n (%) | 278 (34.0) | 194 (32.6) | 84 (37.8) | 0.156 |
| Hypertension, n (%) | 559 (68.3) | 397 (66.6) | 162 (73.0) | 0.082 |
| Diabetes, n (%) | 380 (46.5) | 260 (43.6) | 120 (54.1) | 0.008 |
| Dyslipidemia, n (%) | 615 (75.2) | 439 (73.7) | 176 (79.3) | 0.980 |
| Previous stroke, n (%) | 87 (10.6) | 59 (9.9) | 28 (12.6) | 0.263 |
| **Laboratory examination** |  |  |  |  |
| TC (mg/dl) | 128.59±35.93 | 127.20±35.53 | 132.33±36.79 | 0.070 |
| TG (mg/dl) | 108.50 (80.60-147.91) | 103.63 (79.71-140.83) | 121.34 (84.14-175.37) | <0.001 |
| HDL (mg/dl) | 40.44±9.65 | 41.08±9.71 | 38.74±9.30 | 0.002 |
| LDL (mg/dl) | 71.20±28.60 | 69.67±27.89 | 75.30±30.10 | 0.012 |
| UA (μmol/l) | 305.84±78.81 | 302.14±76.40 | 315.77±84.316 | 0.028 |
| FBG (mg/dl) | 95.76 (85.68-112.68) | 94.05 (84.78-110.30) | 99.99 (88.38-118.26) | <0.001 |
| Scr (μmol/l) | 72.17±16.55 | 70.81±15.16 | 75.83±19.28 | <0.001 |
| eGFR (ml/min/1.73 m^2^) | 92.00±19.79 | 93.26±18.75 | 88.59±22.04 | 0.005 |
| ALB (g/l) | 43.80 (41.80-46.00) | 43.90 (41.93-46.00) | 43.80 (41.38-46.00) | 0.373 |
| LVEF (%) | 62 (57-66) | 62 (57-66) | 62 (57-66) | 0.319 |
| **Medications, n (%)** |  |  |  |  |
| Beta-blockers, n (%) | 461 (56.4) | 351 (58.9) | 110 (49.5) | 0.017 |
| ACEI/ARB, n (%) | 278 (34.0) | 201 (33.7) | 77 (34.7) | 0.797 |
| Aspirin, n (%) | 782 (95.6) | 566 (95.0) | 216 (97.3) | 0.148 |
| Clopidogrel, n (%) | 594 (72.6) | 450 (75.5) | 144 (64.9) | 0.002 |
| Ticagrelor, n (%) | 171 (20.9) | 107 (18.0) | 64 (28.8) | <0.001 |
| DAPT, n (%) | 732 (89.5) | 530 (88.9) | 202 (91.0) | 0.392 |
| Statin, n (%) | 768 (93.9) | 561 (94.1) | 207 (93.2) | 0.639 |
| Dapagliflozin, n (%) | 174 (21.3) | 85 (14.3) | 89 (40.1) | <0.001 |
| **Angiographic characteristics** |  |  |  |  |
| LM-LAD, n (%) | 640 (78.24) | 460 (77.18) | 180 (81.08) | 0.268 |
| LCX, n (%) | 139 (16.99) | 92 (15.44) | 47 (21.17) | 0.066 |
| RCA, n (%) | 450 (55.01) | 329 (55.20) | 145 (53.31) | 0.762 |
| Multi-vessel lesion, n (%) | 124 (45.42) | 129 (47.25) | 121 (54.50) | 0.921 |
| Stent number | 2.33 ± 0.54 | 2.31±0.53 | 2.40 ±0.55 | 0.031 |
| Stent length (mm) | 27.00 (22.00-33.00) | 25.00 (20.00-33.00) | 32.00 (25.00-36.00) | <0.001 |
| Stent diameter(mm) | 3.00 (2.50-3.00) | 2.75 (2.50-3.00) | 3.00 (2.75-3.00) | <0.001 |
| Bare metal stent, n (%) | 44 (5.4) | 29 (4.9) | 15 (6.8) | 0.286 |
| Drug-eluting stent, n (%) | 776 (94.9) | 569 (95.5) | 207 (93.2) | 0.199 |
| Coronary angiography interval  (months) | 26.50 (7.00-64.25) | 24.00 (6.00-63.00) | 32.00 (8.25-69.00) | <0.001 |
| **Coronary artery disease, n (%)** |  |  |  |  |
| SA | 94 (11.5) | 83 (13.9) | 11 (5.0) | <0.001 |
| UA | 628 (76.8) | 468 (78.5) | 160 (72.1) | 0.052 |
| AMI | 96 (11.7) | 45 (7.6) | 51 (23.0) | <0.001 |
| METS-IR | 40.97±6.86 | 40.19±6.29 | 43.05±7.84 | <0.001 |

Notes: Data are presented as mean ± SD, median (IQR), or n (%), as appropriate. A two-sided *p* < 0.05 was considered statistically significant.

Abbreviations: BMI, body mass index; ACEI, angiotensin-converting enzyme inhibitors; ARB, angiotensin receptors; DAPT, dual antiplatelet therapy; DBP, diastolic blood pressure; TC, total cholesterol; TG, triglyceride; FPG, fasting blood glucose; HDL-C, high-density lipoprotein cholesterol; ISR, in-stent restenosis; LDL-C, low-density lipoprotein-cholesterol; LVEF, left ventricular ejection fraction; SBP, systolic blood pressure; UA, uric acid; CR, creatinine; ACEI, angiotensin-converting enzyme inhibitors; ARB, angiotensin receptor blockers; SA, stable angina; UA, unstable angina; AMI, acute myocardial Infarction; METS-IR, metabolic score for insulin resistance.

**Table S4 Significant predictors of ISR >50% and** **ISR >70% in univariate logistic regression analyses**

| **ISR >50%** | | | | **ISR >70%** | | | |
| --- | --- | --- | --- | --- | --- | --- | --- |
| **Variables** | **OR** | **95% CI** | ***p*-value** | **Variables** | **OR** | **95% CI** | ***p*-value** |
| Age | 1.001 | 0.987-1.016 | 0.851 | Age | 1.009 | 0.993-1.026 | 0.261 |
| Male | 0.707 | 0.516-0.968 | 0.031 | Male | 0.829 | 0.590-1.165 | 0.279 |
| BMI | 1.071 | 1.024-1.120 | 0.003 | BMI | 1.082 | 1.031-1.135 | 0.001 |
| LVEF | 0.478 | 0.109-2.093 | 0.327 | LVEF | 0.352 | 0.063-1.981 | 0.236 |
| Smoking | 0.739 | 0.552-0.990 | 0.043 | Smoking | 0.880 | 0.640-1.208 | 0.428 |
| Drinking | 0.689 | 0.512-0.928 | 0.014 | Drinking | 0.785 | 0.569-1.082 | 0.140 |
| Hypertension | 0.689 | 0.503-0.942 | 0.020 | Hypertension | 0.745 | 0.530-1.049 | 0.092 |
| Diabetes | 0.703 | 0.529-0.936 | 0.016 | Diabetes | 0.666 | 0.488-0.908 | 0.010 |
| Previous Stoke | 1.052 | 0.661-1.672 | 0.831 | Previous Stoke | 0.756 | 0.468-1.220 | 0.252 |
| FPG | 1.007 | 1.002-1.011 | 0.005 | FPG | 1.007 | 1.002-1.012 | 0.003 |
| TC | 1.006 | 1.002-1.010 | 0.006 | TC | 1.004 | 1.000-1.008 | 0.066 |
| TG | 1.005 | 1.002-1.007 | <0.001 | TG | 1.005 | 1.003-1.007 | <0.001 |
| LDL-C | 1.008 | 1.003-1.013 | 0.001 | LDL-C | 1.007 | 1.001-1.012 | 0.012 |
| HDL-C | 0.988 | 0.973-1.003 | 0.107 | HDL-C | 0.975 | 0.959-0.992 | 0.975 |
| UA | 1.002 | 1.000-1.004 | 0.051 | UA | 1.002 | 1.000-1.004 | 0.033 |
| Serum creatinine | 1.017 | 1.008-1.026 | <0.001 | Serum creatinine | 1.018 | 1.009-1.027 | <0.001 |
| Beta-blockers | 1.471 | 1.104-1.959 | 0.008 | Beta-blockers | 1.440 | 1.056-1.963 | 0.021 |
| Aspirin | 0.751 | 0.364-1.548 | 0.437 | Aspirin | 0.527 | 0.216-1.285 | 0.159 |
| Clopidogrel | 2.586 | 1.887-3.544 | <0.001 | Clopidogrel | 1.637 | 1.173-2.285 | 0.004 |
| Statins | 1.645 | 0.927-2.921 | 0.089 | Statins | 1.169 | 0.625-2.186 | 0.624 |
| ACEI/ARB | 0.976 | 0.723-1.317 | 0.873 | ACEI/ARB | 0.976 | 0.705-1.351 | 0.882 |
| Stent length | 1.095 | 1.071-1.118 | <0.001 | Stent length | 1.087 | 1.063-1.112 | <0.001 |
| Stent diameter | 2.434 | 1.667-3.553 | <0.001 | Stent diameter | 2.557 | 1.715-3.812 | <0.001 |
| METS-IR | 1.047 | 1.024-1.069 | <0.001 | METS-IR | 1.057 | 1.033-1.082 | <0.001 |
| Standardization of METS-IR | 1.366 | 1.180-1.581 | <0.001 | Standardization of METS-IR | 1.463 | 1.249-1.713 | <0.001 |

Notes: The *p*-values in bold are all <0.05.

Abbreviations: BMI, body mass index; TC, total cholesterol; TG, triglyceride; FBG, fasting blood glucose; HDL-C, high-density lipoprotein cholesterol; ISR, in-stent restenosis; LDL-C, low-density lipoprotein-cholesterol; LVEF, left ventricular ejection fraction; SBP, systolic blood pressure; UA, uric acid; SCr, serum creatinine; ACEI, angiotensin-converting enzyme inhibitors; ARB, angiotensin receptor blockers.

**Table S5 Significant predictors of ISR >50% in multivariate logistic regression analyses**

| **METS-IR index** | **OR (95% CI)** | | |
| --- | --- | --- | --- |
|  | **Model 1** | **Model 2** | **Model 3** |
| Per unit increase | 1.05 (1.02-1.07) ** | 1.04 (1.02-1.07) ** | 1.03 (1.01-1.06) * |
| Per SD increase | 1.36 (1.17-1.59) ** | 1.32 (1.13-1.55) ** | 1.28 (1.02-1.45) * |
| Tertile 1 | 1 Reference) | 1 Reference) | 1 Reference) |
| Tertile 2 | 1.30 (0.90-1.87) | 1.24 (0.86-1.79) | 1.15 (0.77-1.71) |
| Tertile 3 | 1.68 (1.17-2.42) * | 1.53 (1.05-2.23) * | 1.27 (0.84-1.92) |
| *P* for trend | 0.006 | 0.029 | 0.265 |

Model 1: adjusted for age, gender;

Model 2: adjusted for variables with a *p*-value < 0.05 in the univariate analysis, including, smoking, drinking, hypertension, provious storke as well as age and gender;

Model 3: adjusted for age, gender, smoking, drinking, hypertension, diabetes, previous storke, coronary angiography interval, aspirin, total cholesterol, low-density lipoprotein cholesterol, uric acid, LM-LAD lesion, RCA lesion, stent length and stent diameter.

* *p* < 0.05

** *p* < 0.001

The *p*-values in bold are all <0.05.

**Table S6 Predictive value of METS-IR**

| **Variable** | **Cutoff value** | **AUC** | **Sensitivity (%)** | **Specificity (%)** | **Youden index** | **95% CI** | **p-value** |
| --- | --- | --- | --- | --- | --- | --- | --- |
| Predictive value of METS-IR index for ISR >50%. | | | | | | | |
| METS-IR | 42.27 | 0.57 | 49.0 | 63.3 | 0.12 | 0.53-0.61 | <0.001 |
| Predictive value of METS-IR index for ISR >70%. | | | | | | | |
| METS-IR | 42.27 | 0.60 | 52.5 | 63.0 | 0.16 | 0.56-0.64 | <0.001 |
| Predictive value of METS-IR index for ISR >50% in patients with high METS-IR. | | | | | | | |
| METS-IR | 46.41 | 0.59 | 65.5 | 51.6 | 0.17 | 0.52-0.66 | 0.011 |
| Predictive value of METS-IR index for ISR >70% in patients with high METS-IR. | | | | | | | |
| METS-IR | 46.80 | 0.63 | 63.7 | 55.6 | 0.19 | 0.55-0.70 | 0.001 |
| Predictive value of METS-IR index for ISR >50% in patients with stable angina. | | | | | | | |
| METS-IR | 37.02 | 0.63 | 80.0 | 47.8 | 0.28 | 0.51-0.75 | 0.049 |
| Predictive value of METS-IR index for ISR >70% in patients with stable angina. | | | | | | | |
| METS-IR | 39.75 | 0.70 | 72.7 | 66.3 | 0.39 | 0.53-0.87 | 0.033 |
| Predictive value of METS-IR index for ISR >50% in patients with unstable angina. | | | | | | | |
| METS-IR | 41.86 | 0.55 | 49.3 | 59.7 | 0.09 | 0.50-0.60 | 0.041 |
| Predictive value of METS-IR index for ISR >70% in patients with unstable angina. | | | | | | | |
| METS-IR | 42.27 | 0.58 | 49.1 | 61.8 | 0.11 | 0.53-0.63 | 0.003 |
| Predictive value of METS-IR index for ISR >50% in patients with acute myocardial infarction. | | | | | | | |
| METS-IR | 42.71 | 0.61 | 62.5 | 62.5 | 0.25 | 0.50-0.72 | 0.066 |
| Predictive value of METS-IR index for ISR >70% in patients with acute myocardial infarction. | | | | | | | |
| METS-IR | 43.13 | 0.58 | 56.9 | 64.4 | 0.21 | 0.46-0.69 | 0.190 |

Notes: The *p*-values in bold are all <0.05.

Abbreviations: AUC, area under curve; CI, confidence Interval; METS-IR, metabolic score for insulin resistance.

**Table S7 Assessment of the goodness-of -fit of models.**

| **Comparison** | **Model 3 without METS-IR** | **Model 3 with METS-IR** | ***p*-value** |
| --- | --- | --- | --- |
| Continuous NRI (95%CI) | Reference | 0.29 (0.14-0.45) | 0.061 |
| IDI (95%CI) | Reference | 0.02 (0.01-0.03) | 0.032 |
| C-statistic (95%CI) | 0.73 (0.69-0.76) | 0.73 (0.70-0.77) | - |
| AIC | 986.3 | 983.3 | - |
| BIC | 1006.3 | 1068.0 | - |
| df | Reference | 1 |  |

Notes: The *p*-values in bold are all <0.05.

Abbreviations: METS-IR, metabolic score for insulin resistance; AIC, Akaike information criterion; BIC, Bayesian information criterion; df, degree of freedom.
